# Evolution of the *Rdr1* TNL-cluster in roses and other Rosaceous species

**DOI:** 10.1186/1471-2164-13-409

**Published:** 2012-08-20

**Authors:** Diro Terefe-Ayana, Helgard Kaufmann, Marcus Linde, Thomas Debener

**Affiliations:** 1 Institute for Plant Genetics, Leibniz University Hannover, Herrenhaeuser Str. 2, Hannover, 30419, Germany

## Abstract

**Background:**

The resistance of plants to pathogens relies on two lines of defense: a basal defense response and a pathogen-specific system, in which resistance (R) genes induce defense reactions after detection of pathogen-associated molecular patterns (PAMPS). In the specific system, a so-called arms race has developed in which the emergence of new races of a pathogen leads to the diversification of plant resistance genes to counteract the pathogens’ effect. The mechanism of resistance gene diversification has been elucidated well for short-lived annual species, but data are mostly lacking for long-lived perennial and clonally propagated plants, such as roses. We analyzed the rose black spot resistance gene, *Rdr1*, in five members of the Rosaceae: *Rosa multiflora*, *Rosa rugosa*, *Fragaria vesca* (strawberry), *Malus x domestica* (apple) and *Prunus persica* (peach), and we present the deduced possible mechanism of R-gene diversification.

**Results:**

We sequenced a 340.4-kb region from *R. rugosa* orthologous to the *Rdr1* locus in *R. multiflora*. Apart from some deletions and rearrangements, the two loci display a high degree of synteny. Additionally, less pronounced synteny is found with an orthologous locus in strawberry but is absent in peach and apple, where genes from the *Rdr1* locus are distributed on two different chromosomes. An analysis of 20 TIR-NBS-LRR (TNL) genes obtained from *R. rugosa* and *R. multiflora* revealed illegitimate recombination, gene conversion, unequal crossing over, indels, point mutations and transposable elements as mechanisms of diversification.

A phylogenetic analysis of 53 complete TNL genes from the five *Rosaceae* species revealed that with the exception of some genes from apple and peach, most of the genes occur in species-specific clusters, indicating that recent TNL gene diversification began prior to the split of *Rosa* from *Fragaria* in the Rosoideae and peach from apple in the Spiraeoideae and continued after the split in individual species. Sequence similarity of up to 99% is obtained between two *R. multiflora* TNL paralogs, indicating a very recent duplication.

**Conclusions:**

The mechanisms by which TNL genes from perennial *Rosaceae* diversify are mainly similar to those from annual plant species. However, most TNL genes appear to be of recent origin, likely due to recent duplications, supporting the hypothesis that TNL genes in woody perennials are generally younger than those from annuals. This recent origin might facilitate the development of new resistance specificities, compensating for longer generation times in woody perennials.

## Background

Plants are constantly challenged by a large number of different pathogens with diverse infection strategies. To avert these attacks, plants use different mechanisms consisting of active and passive defense lines. Among the active defense mechanisms of plants, specific resistance genes (R-genes) are key factors involved in so-called gene-for-gene interactions. Plants harboring a resistance gene recognize specific avirulence (Avr) gene products that characterize particular genotypes of the pathogen [1,2].

Several R-genes have been isolated from a variety of plant species [3]. The majority of R-genes encode nucleotide-binding site (NBS) and leucine-rich repeat (LRR) proteins [2,4,5]. On the basis of their N-terminal domains, the NBS-LRR resistance genes can be subdivided into two classes. The first class encodes proteins with an N-terminal TIR domain (homology to the *Drosophila* Toll and mammalian Interleukin-1 receptors), whereas the second class encodes proteins with coiled-coils (CC), sometimes in the form of a leucine zipper (LZ) at the N-terminus of the protein [3,6,7]. Two basic strategies for pathogen recognition are currently thought to exist: direct recognition of Avr-gene products by R-proteins and indirect recognition via sensing perturbations of host proteins (the so-called guard hypothesis) [2,8]. Different domains of the NBS-LRR R-genes have been shown to be involved in pathogen recognition, but most studies indicate that the LRR domain plays the most important role in pathogen recognition [9].

Most of the R-genes described to date are organized in clusters reviewed in [3,10]. This clustering may facilitate R-gene diversity in the course of adaptation to counteract newly emerging Avr-protein variants in newly evolving virulent races of a pathogen.

Extensive studies have been conducted to understand the mechanism of R-gene diversification, mainly in herbaceous annual plants, such as for *Rp1* in maize [11-15], *Cf4/Cf9* and *Mi-1* in tomato [16-18], *Xa21* in rice [19], *Dm3* (*RGC2*) in lettuce [20-23], *RPP5* in *Arabidopsis*[24], *N* in flax [25] and *R1* in potato [26]. Sequence analyses from these studies indicate that R-genes display significantly higher rates of sequence evolution than other plant genes. Furthermore, LRR domains generally evolve more rapidly than the other domains of NBS-LRR genes and often display signs of positive selection. Tandem and segmental gene duplications, recombination, unequal crossing over, point mutations and diversifying selection have been shown to contribute to R-gene diversity. Recent R-gene sequence analyses in *Arabidopsis*, maize, tomato, barley, lettuce, rice and wheat further indicated illegitimate recombination (IR) as a major source of duplications and deletions [27]. Illegitimate recombination is a type of recombination between two DNA molecules which are not necessarily homologous to each other but share a few identical sequences. These identical sequences are called illegitimate recombination signatures. Illegitimate recombination may result in duplications or deletions [27].

Unlike herbaceous annuals, woody perennial species are characterized not only by long-lived individual plants but also by longer average generation times than annuals. Therefore, the nature of R-gene diversification could vary from that of annual plants. Some perennial plant species, such as roses, propagate clonally as well as sexually via seeds. These different forms of reproduction could also contribute to a possible deviation in R-gene diversity in perennials, as differences in evolutionary rates between annuals and perennials have been noted several times [28]. The mechanisms underlying such differences are still unknown. More frequent and recent duplications of R-genes have been described in poplar and grapevine compared with rice and *Arabidopsis*[29], indicating different evolutionary patterns of R-genes between perennial and annual plants.

Roses are attacked by a number of pathogens and pests [30], among which black spot is the most severe disease of field-grown roses. It is caused by the hemibiotrophic ascomycete *Diplocarpon rosae*, for which a number of pathogenic races have been identified [31]. Resistance to black spot has been found to be caused by both quantitative and qualitative resistance genes [32], with the single dominant R-gene *Rdr1* from *R. multiflora* being the best studied rose R-gene thus far [33].

Recently, *Rdr1* was finely mapped to a telomeric position in rose linkage group 1 in a contig of four overlapping BAC clones and isolated via map-based cloning [33-35]. The *Rdr1* gene is a TIR-NBS-LRR (TNL) type resistance gene and a member of a multigene family of nine highly similar genes clustered in a region of 265.5 kb in *R. multiflora*.

Here, we present sequence information from the *Rdr1* locus of a second rose species, *R. rugosa*, and analyze the sequence conservation of this locus and the TNL family within roses. Furthermore, we analyze synteny with other *Rosaceae*, represented by sequences from strawberry, peach and apple, and with members of other plant families.

## Results

### Comparison of the *Rdr1* contig between *R. multiflora* and *R. rugosa*

In addition to the previously published sequence of a 265.5-kb region spanning the *Rdr1* locus of *R. multiflora*, a set of four overlapping BAC clones spanning the *Rdr1* region in *R. rugosa* was sequenced with Roche 454 sequencing. The sequences were assembled to a total length of 340,415 bp, with individual sizes of 96.3, 144.9, 75.4 and 78.6 kb for the BAC clones 31C14, 95G17, 78F5 and 35D6, respectively. The complete sequence has been deposited in GenBank [accession number GenBank: JQ791545]. The first 67,036 bp from the *R. rugosa* sequence extended beyond the left end of the corresponding *R. multiflora* homologous BAC-clone 29O3.

**Figure 1:**
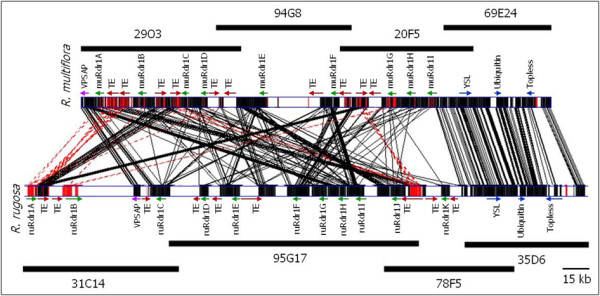
**The complete contig alignment and schematic representation of the predicted TNL genes, transposable elements (TE) and other conserved genes in the *Rdr1 *region in *R. multiflora *and * R. rugosa *.** Partial gene annotation is indicated by arrows. The TNL genes are represented with green arrows. The transposable elements (TE) are represented with red arrows. Vacuolar protein sorting-associated protein (VPSAP) at the left border of *R. multiflora* contigs are represented with purple arrow and the highly conserved genes at the right part are represented with blue arrows. Similar sequences are connected by black lines. Similar sequences but in reverse orientation are connected with red lines. Unfilled boxes represent sequence absent in one or another contig. The region around TNL and TE displays copy number changes, inversions and deletions/insertions. The overlapping horizontal black lines represent the respective BAC clones that were sequenced to assemble the contigs. GATAligner with default parameters was used for the alignment. GATAPlotter parameters with min: 1E^-5^ were used to plot the graph.

Ab initio gene prediction revealed 65 protein-coding genes, 46 of which displayed significant similarities to entries in the GenBank database (Table 1). Among the 65 predicted genes, eleven are TNL genes with a high DNA sequence similarity (88% to 95%) to the already characterized *Rdr1* TNL family, and nine are transposable elements (Figure 1). We designated the 11 TNL genes as *ruRdr1* A through *ruRdr1*K, where ‘ru’ stands for the species name of the source of the sequence (*R. rugosa*), and ‘A’ to ‘K’ indicates the order of the TNL gene in the sequenced contig. A total of 10 of the 11 TNL sequences exhibit uninterrupted predicted coding sequences. We also observed several truncated TNL genes distributed throughout the cluster. Most of these fragments share similarities to LRR domains, and some are attached to retroelements. Six of the transposable elements belong to the group of long terminal repeat (LTR) retrotransposons; two belong to the non-long terminal repeats (non-LTRs) retrotransposons; and one belongs to the mutator-like transposase type of DNA transposons.

**Table 1:** **List of predicted genes from the 340,415-bp contig of *R. rugosa *orthologous to the *Rdr1 *locus of *R. multiflora***

**No.**	**Position on the contig (bp)**	**Similarity as revealed by BLASTp (similar to GenBank accession number)**	**E-value**
1	6564-11898	TIR-NBS-LRR (AEE43932.1)	0.0
2	12848-11923	None	-
3	12986-18853	Retrotransposon protein, Ty1-copia (ABF96803.1)	0.0
4	20854-26772	Retrotransposon protein, Ty1-copia (ABA98286.2)	0.0
5	28978-37792	TIR-NBS-LRR (AEE43932.1)	0.0
6	41656-37934	None	-
7	45166-56998	Neuroblastoma-amplified sequence (XP_003602296.1)	0.0
8	60939-57122	Major facilitator superfamily domain (XP_003526731.1)	0.0
9	62610-61549	None	-
10	62646-64390	rhodanese-like domain-containing protein (NP_567785.1)	1e^-102^
11	68835-64533	Vacuolar protein sorting-associated (XP_002274585.1)	0.0
12	69119-71844	Transcription factor B3 (ABN06173.1)	7e^-15^
13	71912-76631	Gag-pol polyprotein (BAK64102.1)	0.0
14	76951-79409	Transcription factor B3 (XP_003517920.1)	2e^-19^
15	85078-79980	TIR-NBS-LRR (AEE43932.1)	0.0
16	88492-94115	None	-
17	94486-100495	None	-
18	104305-100835	Mutator-like transposase (BAB10320.1)	1e^-102^
19	109120-104792	TIR-NBS-LRR (AEE43932.1)	0.0
20	109935-111490	None	-
21	116498-111549	Gag-pol polyprotein (AAO73527.1)	2e^-55^
22	121170-117613	Shikimate dehydrogenase (EEF45470)	6e^-12^
23	127179-122701	TIR-NBS-LRR (AEE43932.1)	0.0
24	127707-139272	Non-LTR retroelement reverse transcriptase (AAG13524)	0.0
25	140854-139893	None	-
26	143524-147207	Transcription factor B3 (XP_003535137.1)	2e^-08^
27	148930-147755	Phospholipase C (ACF93733.1)	6e^-12^
28	153630-150256	None	-
29	161422-157057	TIR-NBS-LRR (AEE43932.1)	0.0
30	166328-163477	LRR (AEE43932.2)	4e^-85^
31	169607-170212	None	-
32	176508-171812	TIR-NBS-LRR (AEE43932.1)	0.0
33	176830-179287	Transcription factor B3 (XP_003517920.1)	2e^-21^
34	179594-182362	Transcription factor B3 (XP_003517920.1)	3e^-18^
35	188265-182622	TIR-NBS-LRR (AEE43932.1)	0.0
36	198290-192614	TIR-NBS-LRR (AEE43932.1)	0.0
37	199678-207935	None	-
38	211387-209495	None	-
39	217944-212928	TIR-NBS-LRR (AEE43932.1)	0.0
40	230339-218090	Retrotransposon protein, Ty1-copia (ABF96803.1)	0.0
41	232191-238449	Non-LTR retroelement reverse transcriptase (AAB82639)	0.0
42	239180-238929	None	-
43	239877-240955	None	-
44	245771-241137	TIR-NBS-LRR (AEE43932.1)	0.0
45	250194-245924	Copia-type polyprotein (AAG51247.1)	0.0
46	250788-253960	None	-
47	254886-254010	None	-
48	258861-257151	ATP binding protein (XP_002515676.1)	0.0
49	265170-261078	AAA domain-containing protein (XP_003544721.1)	0.0
50	266721-274192	Yellow stripe-like protein (XP_003602315.1)	0.0
51	278098-274211	GTPase-activating protein (XP_003526739.1)	0.0
52	283722-281796	6-phosphogluconolactonase (XP_002518214.1)	5e^-135^
53	285094-286783	Ubiquitin (XP_002530306.1)	4e^-88^
54	288959-286931	Aldo-keto reductase (XP_003602320.1)	0.0
55	290639-289124	Homeobox leucine zipper protein (AAD38144.1)	1e^-79^
56	298129-297002	None	-
57	298247-299808	Hypothetical protein (XP_003602325.1)	5e^-97^
58	309056-299987	TOPLESS-RELATED protein (XP_002275116.1)	4e^-105^
59	309339-311499	None	-
60	318648-317787	None	-
61	325145-319916	Serine/threonine protein kinase (NP_001234146.1)	0.0
62	326294-328526	None	-
63	331756-329732	UDP-N-acetylglucosamine transporter (XP_003531350.1)	5e^-48^
64	335561-332977	F-box protein (XP_003610959.1)	2e^-08^
65	336756-340415	Unnamed protein product (CBI23069.3)	0.0

Gene predictions were made using FGENESH and BLAST.

**Figure 2:**
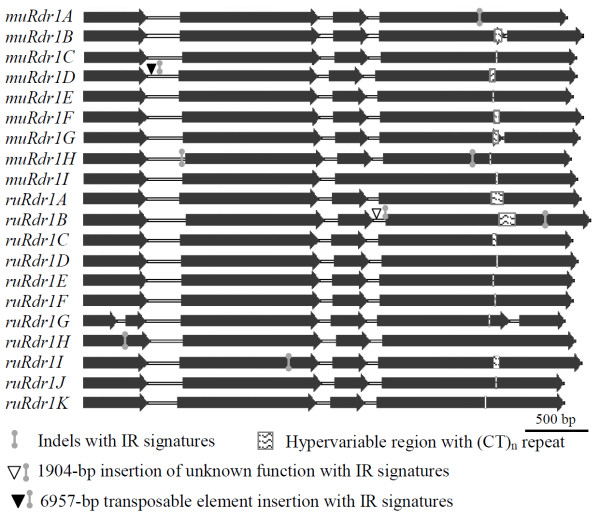
**The intron-exon structures of the 20 *Rdr1 *homologs from roses.** The majority of the *Rdr1* genes include four exons and three introns with few homologs having five exons that correspond to the TIR, NBS, NLL (NBS-LRR linker), LRR and post-LRR domains, respectively. Several illegitimate recombination (IR) signatures are distributed across the sequence, flanking indels or repeats. The LRR region is characterized by variable number of (CT)_n_ repeats ranging from zero in *muRdr1A* and *ruRdr1H* to 65 in *ruRdr1B*[41].

Similar to the previously described *R. multiflora* TNLs [35], the size of the genomic sequences of the *R. rugosa* TNLs varies from 4329 bp to 5677 bp. The *ruRdr1B* TNL carries an additional 1904-bp sequence of unknown function in the third intron (Figure 2). Most of the TNL homologs are comprised of four exons and three introns with few homologs having five exons that correspond to the TIR, NBS, NLL (NBS-LRR linker), LRR and post-LRR domains, respectively (Figure 2).

The additional sequence that extends the *R. rugosa* contig beyond the borders of the *R. multiflora* contig contains two TNL elements (*ruRdr1A* and *ruRdr1B*) as well as two transposable elements and sequences with similarity to a neuroblastoma amplified gene, a major facilitator superfamily domain and a rhodanese-like domain-containing protein.

GATA alignment and dot plot comparison of the 265.5-kb region from *R. multiflora* and the 340.4-kb region from *R. rugosa* indicates a high degree of synteny between the two species (Figure 1).

A group of nine sequences (ATP binding, AAA type ATPase, Yellow stripe-like, GTPase activator, 6-phosphogluconolactonase, Ubiquitin fusion, Homeobox leucine zipper, TOPLESS-RELATED and Serine/threonine protein kinase) at the right end of the *R. multiflora* contig and a sequence stretch comprising a predicted gene for a vacuolar protein sorting-associated protein and transcription factor at the left end of the contig are perfectly conserved between the two species. However, the region between these sequences exhibits several copy number changes, inversions and deletions/insertions. Of the 11 TNL genes located on the *R. rugosa* contig, nine are in the same and two are in a reverse orientation compared with the *R. multiflora* contig in which all of the TNL genes are in the same orientation. For some of the non-TNL genes, differences are observed in terms of the relative location and the number of homologs within each cluster. Furthermore, some sequences are completely missing in one cluster and present in another. For example, a 23-kb region with similarity to prolyl 4-hydroxylase alpha, aminotransferase-like, WUSCHEL protein terminator and inosine-5'-monophosphate dehydrogenase is present in the homologous locus in *R. multiflora* but absent in the *R. rugosa* locus.

In addition, the transposable elements distributed over the two contigs differ both in their position and sequence. The ten transposable elements in the *R. multiflora* locus belong to the *Ty1/copia* type retroelements, whereas the *R. rugosa* locus contains Ty1/copia as well as other different retroelements.

### Conservation of the locus within the *Rosaceae*

Among the plant species with sequenced genomes, the strawberry is the closest relative to the genus *Rosa*. We therefore compared the *R. multiflora* contig to sequences from *Fragaria vesca*. We subjected individual sequences from the *Rdr1* contig to BLAST searches against the *Fragaria* genome sequence and located a stretch of similar sequences of approximately 354 kb from strawberry chromosome 7 between positions 19,798,478 bp and 20,152,477 bp. Several insertions, deletions and large rearrangements in the form of inversions and translocations were found (Additional file 1). The cluster of conserved genes from the right and the left sides of the *R. multiflora* contig are also found in *Fragaria*. Twelve TNL genes with high similarity to the *Rdr1* gene family (71% to 87% identity at the DNA level and 61% to 81% similarity at the amino acid level) are also located in the selected *Fragaria* sequence region. In the following analyses, we designate these genes as FvTNL1 through FvTNL12. Unlike at the *R. multiflora* locus, the orientation of the *Fragaria* TNLs varies, in that the majority of the genes are inverted relative to the *R. multiflora* copies. Further differing from the *R. multiflora* locus, the genes within the conserved cluster from the right side of the contig, such as the yellow stripe-like gene and the ubiquitin fusion gene, are in an inverted position, inserted within the TNL genes.

The 354-kb *Fragaria* sequence contains a stretch of 14.2 kb of ambiguous sequence (represented by stretches of Ns) resulting from problems in the assembly. This may lead to changes in the *Fragaria* locus structure in the future, although this is unlikely.

In contrast to what is observed in *Fragaria*, the *Rdr1* homologous locus is located on two different chromosomes in *P. persica* (Additional file 2). The closest relatives to the *Rdr1* gene family are found in a cluster of 15 genes in linkage group 8 (scaffold no. 8 from bases 2,050,000 bp to 2,510,000 bp), whereas the genes flanking the TNL cluster at the right and left margins in *Rosa* and *Fragaria* are located in linkage group 2 (scaffold no. 2 from 26,050,000 bp to 26,110,000 bp) in *Prunus*. The 15 *Prunus* TNLs are designated PpTNL1 through PpTNL15. The cluster is characterized by large differences in terms of the non-TNL genes and the orientation of the TNLs. In contrast, the flanking genes are highly conserved between *Rosa* and *Prunus*.

The similarity between rose and apple is comparable to the above-mentioned situation in peach, in which TNLs and flanking genes are located in two different linkage groups (Additional file 3). The closest relatives to the *Rdr1* TNLs are located in a cluster of 11 genes in apple linkage group 15 spanning a position from 41,166,396 bp to 41,719,891 bp. Hereafter, they are designated MdTNL1 through MdTNL11. The flanking genes are located in linkage group 1 (position 35,200,000 bp to 35, 294,999 bp).

### Conservation of the locus in taxa outside of the *Rosaceae*

The strong synteny of the *Rdr1* locus between *Rosa* and *Fragaria* and the low synteny with *Prunus* and *Malus* raise the question of whether the group of *Rdr1* TNL genes is present at a similar locus in other plant families. We defined synteny simply as close linkage of a TNL cluster with similarity to the *Rdr1* family to the flanking genes conserved among the Rosoideae species.

The first species investigated was *Medicago truncatula*, as the *Fabaceae* are a family closely related to the *Rosaceae*. We found some of the flanking genes of the right side of the contig distributed among more than three chromosomes in *M. truncatula*, but no TNL genes related to the *Rdr1* family are located close to that locus (Additional file 4). We also did not detect the vacuolar protein sorting-associated protein or transcription factor from the left side of the *R. multiflora* locus, indicating a lack of synteny in *Medicago*. Related TNL genes are found on several of the *Medicago* chromosomes, but the similarity to the *Rdr1* family is too low to infer orthology relationships. The *M. truncatula* sequences utilized in these analyses are downloaded from http://www.medicagohapmap.org/.

We performed the same analysis in the *Arabidopsis thaliana* genome, and again, no syntenic block of sequences could be detected (Additional file 5). The flanking genes in this case also do not form a cluster at one location but are distributed on more than two chromosomes in more than two copies. The *A. thaliana* sequences utilized in these analyses are downloaded from http://www.arabidopsis.org/.

### Evolutionary history of the *Rdr1* TNL family

**Figure 3:**
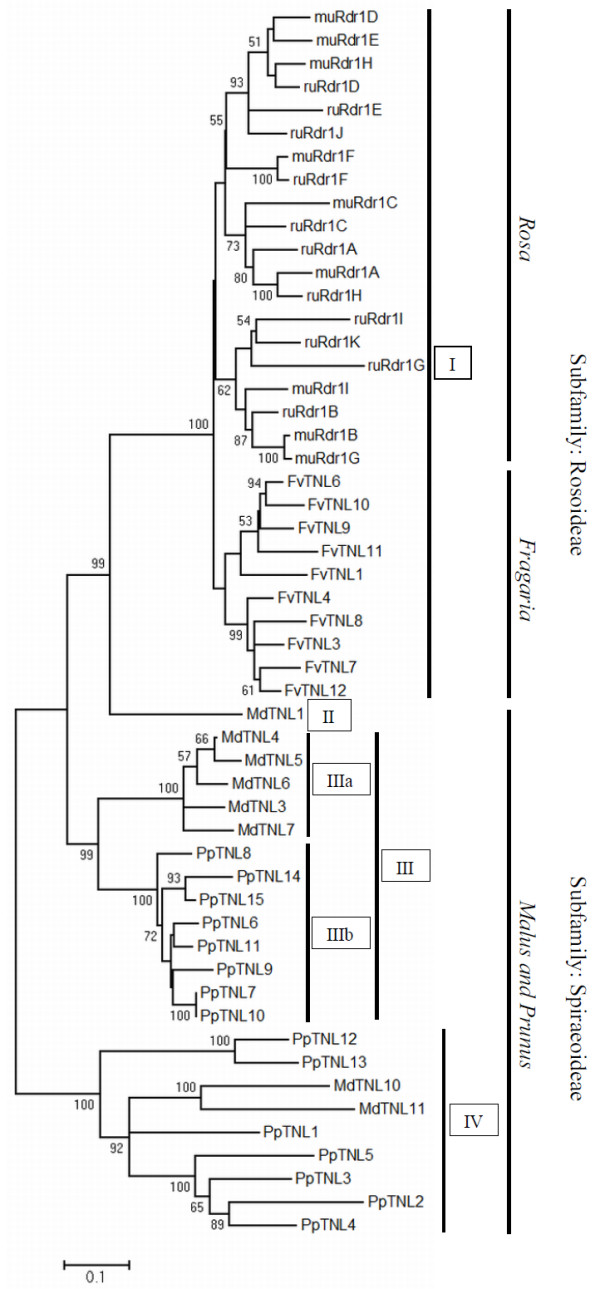
**Phylogenetic analysis of the amino acid sequences of the 53 TNLs.** The evolutionary history was inferred using the Maximum Likelihood method based on the JTT matrix-based model. The bootstrap consensus tree inferred from 500 replicates is taken to represent the evolutionary history of the sequences analyzed. Branches corresponding to partitions reproduced in less than 50% of bootstrap replicates are collapsed. The two subfamilies, Rosoideae and Spiraeoideae, are clearly separated. The TNLs from the genera *Rosa* and *Fragaria* are also separate. Intermixed branching is shown for the 20 *Rdr1*-TNLs from *R. multiflora* and *R. rugosa*. The 23 TNLs of apple and peach are clustered in multiple clades or subclades. The TNLs for each clade or subclade are species specific, with few intermixing. The tree is drawn to scale, with branch lengths measured in terms of the number of substitutions per site. Bootstrap values of more than 50 are indicated next to the branches.

On the basis of our first analyses of the conservation of the structure of the *Rdr1* locus, we performed a phylogenetic analysis of the TNL genes from the *R. multiflora*, *R. rugosa* and *F. vesca* contigs. In addition, we included all 15 peach and 11 apple genes clustered in linkage groups 8 and 15, respectively. Overall, the derived amino acid sequences of 53 full-length TNL genes were aligned (three truncated genes from apple and two from *Fragaria* were discarded because they did not contain TIR, NBS and LRR domains), and a maximum likelihood phylogenetic tree was constructed with 500 bootstrap replicates (Figure 3).

The tree shows four major groups that are highly supported by bootstrap values of 99% to 100%. There is a distinct cluster (I) formed by *Rosa* and *Fragaria*, representing the subfamily Rosoideae of the *Rosaceae*, which is separated from a single *Malus* sequence (MdTNL1, II), a cluster (III) in which sequences from *Malus* and *Prunus* each form distinct subclusters and a cluster (IV) comprised mainly of *Prunus* sequences and two *Malus* sequences in one subcluster (Figure 3). Within the largest Rosoideae cluster, sequences from *Rosa* and *Fragaria* each form distinct highly supported subclusters, indicating recent evolution of the *Rdr1* TNLs after the genera diverged. Within the subcluster comprised of the rose sequences, there is no separation of *R. multiflora* and *R. rugosa* sequences. Instead, highly supported clusters with pairs of sequences from both species (e.g., *muRdr1F*/*ruRdr1F*, *muRdr1A*/*ruRdr1H*) and mixed subclusters with low bootstrap support indicate that some of the sequences evolved before the species separated. As an exception to this, the pair *muRdr1B*/*muRdr1G* shows almost no divergence, indicating a recent gene duplication in *Rosa multiflora*.

In contrast to the divergence of sequences from *Rosa* and *Fragaria*, the *Rdr1* homologues in *Prunus* and *Malus* are mixed in clusters III and IV. Cluster III is comprised of two subclusters (IIIa and IIIb), each including genes only from *Prunus* or *Malus*, in contrast to cluster IV, which consists of genes from both species. The branch lengths of subclusters IIIa and IIIb are shorter than the branch lengths of cluster IV, indicating that they evolved after *Prunus* and *Malus* diverged.

### Sequence evolution of the TNL family

To obtain additional information about the processes that led to the diversity of the *Rdr1* TNL genes, we analyzed the sequence variability of the genes in more detail.

**Figure 4:**
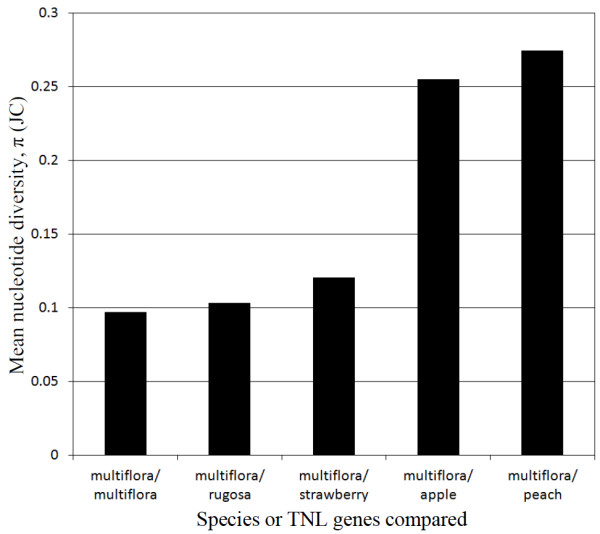
**Average nucleotide diversity values (π) within *R. multiflora Rdr1 *-TNL genes and between *R. multiflora *and four other species of the *Rosaceae.***

**Figure 5:**
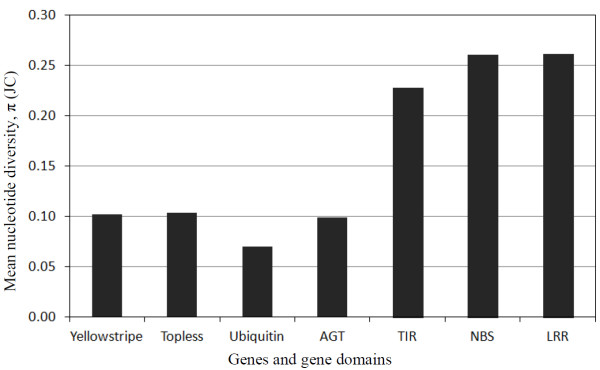
**Average nucleotide diversity between the 53 *Rosaceae Rdr1 *-TNLs and between the three flanking genes as well as the AGT gene.** The *Rosaceae* TNLs and their domains are characterized by larger mean π values than the flanking genes and AGT (alanine:glyoxylate aminotransferase). AGT is present as an arbitrarily selected gene, which occurs as a single copy gene in most plant genomes.

Analyses of nucleotide diversity (π) among the TNL genes from *Rosa* ranged from 0.0046 between the closely related paralogs mu*Rdr1*B and mu*Rdr1*G (DNA identity 99%) to 0.1413 between mu*Rdr1*F and ru*Rdr1*G (Figure 4). The nucleotide diversity within the cluster of *R. multiflora* TNL genes is at approximately the same level as the average for the *R. multiflora* genes compared with the genes from *R. rugosa*. Surprisingly, comparison of all *R. multiflora* TNL genes with the genes from the *F. vesca* cluster leads to only slightly higher values (all approximately 0.1) than comparisons among the rose genes. In contrast, a comparison with the other two rosaceous genomes (*Malus* and *Prunus*) leads to a sharp increase in the diversity, associated with an average π value of 0.2492 (Figure 4). If TNL and non-TNL genes from the orthologous regions are compared, the value is much higher than the average value for the conserved cluster of flanking genes (π = 0.1) or the value for a randomly selected single-copy control gene (AGT, alanine:glyoxylate aminotransferase) from an EST collection (π = 0.099). This indicates higher evolutionary rates for the TNL genes compared with other genes from the same locus or unlinked loci (Figure 5). The large variation in sequence similarity between the 53 *Rosaceae* TNLs obscured the obvious difference in diversity and led to a similar level of nucleotide diversity for the TIR, NBS and LRR domains (Figure 5). A clear difference is observed between the TIR, NBS and LRR domains when the more similar TNL genes from *R. multiflora*, *R. rugosa* and /or *Fragaria* are compared (Additional file 6), which is in line with previous reports in which the TIR domain is the most conserved region, and the NBS and LRR domain regions are more diverse.

**Figure 6:**
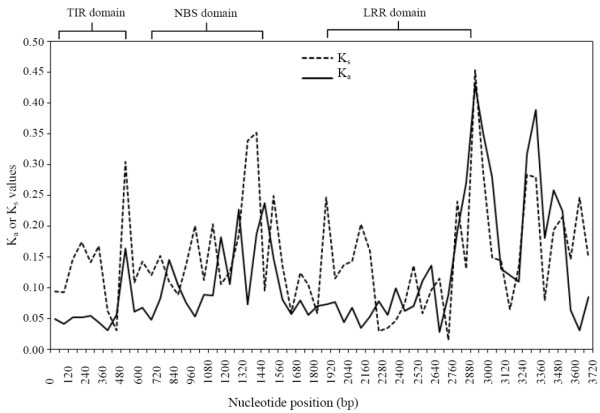
**Sliding window analyses of synonymous and nonsynonymous substitutions in different regions of the 20 *Rdr1 *-TNLs sequences from *R. multiflora *and *R. rugosa *.** Low Ka/Ks ratios (<1.0) dominate the entire sequence, but few regions with elevated Ka/Ks ratios (>1.0) can be found, increasing in number towards the LRR region.

As several studies on the evolution of TNL genes in plants have indicated positive selection acting on the LRR region, we computed Ka and Ks values for the whole coding region of all 20 rose TNL genes. The overall mean Ks values estimated using the modified Nei and Gojobori algorithm [36] with Jukes-Cantor correction [37] are 0.12, 0.150 and 0.11 for the TIR, NBS and LRR domains, respectively. Whereas the overall mean Ka estimates are 0.06, 0.10 and 0.08 for the TIR, NBS and LRR domains, respectively. Recalculation over short sequences by dividing each of the domains into two to four parts revealed elevated Ka over Ks values for a few pairwise comparisons, particularly in the LRR region, which is indicative of positive selection (Figure 6). The number of comparisons with an elevated Ka over Ks increases from the TIR to NBS domains, and the highest numbers occurred in the LRR domain region (Figure 6). This is in agreement with several previous studies, in which different ratios of Ka to Ks were found for the different subunits of the LRR domains, with the highest ratio of Ka to Ks observed for the solvent-exposed residues of the LRR subunit, which are speculated to determine resistance specificity [16,38]. The smallest Ks and Ka values are 0.012 and 0.005, respectively, detected between the most similar *Rdr1* paralogs, *muRdr1B* and *muRdr1G*.

**Figure 7:**
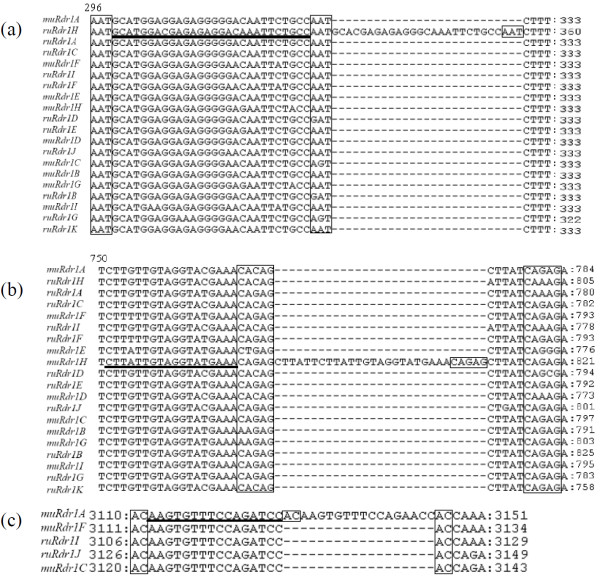
**Illegitimate recombination (IR) signatures flanking different regions of the 20 *Rdr1 *-TNL homologs. a**) IR-flanking TIR domain. **b**) IR-flanking NBS domain. **c**) IR-flanking LRR domain. Two to five base IR signatures are framed. The sequence region with random duplication is underlined. Certain sequence parts of the IR signatures are imperfect with few mismatches. In each case, duplication of at least one sequence is visible following the IR signature.

Comparison of the 20 *R. multiflora* and *R. rugosa* TNLs revealed several duplications and deletions distributed along the entire domain of the gene. Similar to previous observations related to R-gene sequences in annual plant species [27], illegitimate recombination (IR) signatures are detected flanking eight duplications and indels (Figure 2). For example, an 'AAT' fingerprint flanked the 27-bp repeat in the TIR-encoding domain of *ruRdr1H*; a 'CAGAG' fingerprint flanked the 18-bp repeat in the NBS-encoding domain of *muRdr1H*; and an 'AC' fingerprint flanked the 16-bp repeat in the LRR-encoding domain of *muRdr1A* (Figure 7). In most cases, the IR signatures are 100% identical.

Approximately 1270 polymorphic sites and 87 indels varying in length from 1 bp to 160 bp are observed. The longest indel is in the microsatellite repeat region in exon 4. Some indels are duplications flanked by IR signatures resulting from unequal crossing over. The majority of indels are in the noncoding region of the TNL sequences.

Analyses with Geneconv [39] uncovered 26 gene conversion tracts among the 20 TNLs of *R. multiflora* and *R. rugosa* (Table 2). Tracts of 100% identical sequences are identified as an indication of sequence conversion. Sequence conversion events were visually confirmed in different regions of the TNL genes.

**Table 2:** **The 26 gene conversion tracts detected in the 20 TNL homologs of the two rose species,* R. multiflora *and *R. rugosa***

**TNLs**	**Sequence tract begin (bp)**	**Sequence tract end (bp)**	**Sequence length (bp)**
*ruRdr1H*;*ruRdr1I*	722	956	235
*muRdr1F*;*ruRdr1F*	1515	1800	286
*ruRdr1H*;*ruRdr1J*	1580	1701	122
*muRdr1F*;*ruRdr1F*	1127	1391	265
*ruRdr1A*;*ruRdr1J*	1580	1715	136
*muRdr1F*;*ruRdr1I*	2868	3125	258
*ruRdr1H*;*muRdr1C*	2664	2843	180
*muRdr1H*;*muRdr1B*	1	209	209
*ruRdr1H*;*ruRdr1A*	1552	1701	150
*muRdr1C*;*ruRdr1K*	2823	2995	173
*muRdr1H*;*muRdr1G*	1	205	205
*ruRdr1I*;*ruRdr1J*	2243	2394	152
*ruRdr1H*;*ruRdr1G*	3111	3227	117
*muRdr1F*;*ruRdr1F*	2001	2333	333
*ruRdr1J*;*ruRdr1K*	229	445	217
*muRdr1H*;*muRdr1G*	211	418	208
*muRdr1F*;*ruRdr1F*	182	519	338
*muRdr1F*;*ruRdr1I*	2651	2866	216
*ruRdr1A*;*ruRdr1C*	211	428	218
*muRdr1A*;*muRdr1I*	535	624	90
*ruRdr1C*;*ruRdr1I*	276	453	178
*muRdr1A*;*muRdr1D*	233	416	184
*muRdr1D*;*ruRdr1J*	1704	1839	136
*ruRdr1I*;*ruRdr1K*	1598	1676	79
*muRdr1F*;*ruRdr1I*	3992	4179	188
*muRdr1I*;*ruRdr1G*	1717	1841	125

## Discussion

### The *Rdr1* locus is conserved within the *Rosa* genus

We sequenced a genomic region of 340.4 kb from *R. rugosa* orthologous to the recently published *Rdr1* gene cluster from *R. multiflora*. Comparison of the two regions reveals a high degree of conservation of genes flanking a cluster of 11 TNL genes, but we also found rearrangements within the group of TNLs. This corresponds to other studies in which major rearrangements, including deletions, gene duplications and inversions, have been found among groups of NBS-LRR genes [7]. The structure of the *Rdr1* locus indicates several mechanisms that have led to the above-mentioned structural differences between the two orthologous regions. The close relationships between the TNL genes, presenting DNA similarities with between an 88% and 95% identity, might have led to unequal crossing over, resulting in some of the observed duplications/deletions. Another factor promoting recombination at the *Rdr1* locus is the presence of transposable elements belonging to the *Ty1/Copia* class as well as non-LTR classes, which are present on both contigs. Several authors have hypothesized that both the repetitive nature of various copies of retroposons and their capacity to transpose neighboring genes have likely contributed to the diversity of R-gene clusters [10,40]. The high variability detected among the TNL genes at the locus is also reflected in the large number of alleles for a microsatellite from the LRR region of the *Rdr1* gene family that has been analyzed in rose varieties, species and individuals from natural populations of the diploid species *Rosa arvensis*[41].

### The *Rdr1* locus is conserved between *Rosa* and *Fragaria*

Comparing the rose *Rdr1* locus with a syntenic region of the *Fragaria* genome revealed conservation of the flanking genes as well as the presence of the *Rdr1* TNL gene family. This finding is in agreement with synteny studies conducted with molecular markers showing that the region on rose chromosome 1 to which we mapped *Rdr1* is syntenic to *Fragaria* chromosome 7 [42,43]. It also indicates that a cluster of TNL genes with similarity to the *Rdr1* TNLs existed prior to the separation of the tribe Rosa (to which roses belong) from the Potentilleae (to which strawberries belong). Apart from the flanking genes and the presence of TNLs with high similarity to the *Rdr1* family, large structural changes occurred, in that many non-TNL genes found in rose do not match similar genes in strawberry and vice versa. As the two species belong to separate tribes, this is not surprising, given the variability that we detected between the two rose species. The mechanisms leading to these differences are likely the same mechanisms that shaped the differences within the genus *Rosa*.

### The *Rdr1* locus is not conserved between *Rosa*, *Prunus* and *Malus*

In contrast to the conservation of the *Rdr1* locus in *Fragaria*, no conservation of the TNL cluster is found in *Malus* and *Prunus*. Although the flanking genes are conserved as a tightly linked group in both *Malus* and *Prunus*, there are no closely linked TNLs, as observed in *Fragaria*. Rather, the closest relatives to the rose *Rdr1* family are detected in clusters on separate chromosomes. As conservation is also lacking in *Medicago* and *Arabidopsis*, one possible conclusion is that the TNLs were inserted at their present location after the Rosoideae and Spiraeoideae split. Although errors during whole genome sequencing and assembly cannot be excluded [44], the cause of this transposition is difficult to infer. However, the presence of mobile genetic elements is one possible explanation. Comparative analysis of an NBS-LRR cluster in *Phaseolus vulgaris* and other *Fabaceae* indicates that subtelomeric positions are prone to transpositions of repeated DNA elements [45]. This might be the case for the *Rdr1* locus split in Spiraeoideae, as the *Rdr1* TNL clusters are located at a telomeric position in the rose chromosome 1 map [33]. In contrast to the situation in *Phaseolus*, we have no evidence that any sequence closely related to the *Rdr1* family maps outside the cluster on linkage group 1. Mapping experiments in two diploid and one tetraploid mapping population reveal that all polymorphic fragments are linked in one region on linkage group 1, although they spread over a distance of up to 18 cM (42).

### Phylogenetic analyses

Phylogenetic analyses (Figure 3) led to a dendrogram that was highly supported by bootstrap values above 80%, in which the rose and *Fragaria* genes form separate clusters, whereas the *Malus* and *Prunus* genes are located in mixed clusters. None of the *Fragaria* genes clustered with any of the rose genes, which form clusters according to the two different rose species.

This indicates that although the TNL genes most likely translocated to their current positions after the separation of the Rosoideae and the Spiraeoideae, the locus underwent further independent evolution in each of the genera. This is consistent with the observation of high similarity among the DNA sequences within each cluster, indicating that individual members of the gene families arose relatively recently. Explanations for this situation have been proposed in a number of other studies on R-genes and involve the evolution of R-gene clusters via duplications and deletions of family members and through gene conversion [10]. The discrepancy of TNL genes in woody plants forming clusters of highly similar genes while TNL genes are generally phylogenetically old, predating the split between gymnosperms and angiosperms, has been noted previously [29]. Studies on the sequenced genomes of grapevine and poplar compared with rice and *Arabidopsis* indicate that most clustered TNL genes from woody perennials are of a more recent origin than genes from annual plant species [29]. One explanation for this observation is the lower number of generations in woody perennials compared with annuals, which is also held responsible for the generally lower rate of molecular evolution in protein coding genes [28]. Within cluster I of the dendrogram, the rose subclusters include genes from both species (Figure 3), indicating that these copies existed before the species separated. There are only three exceptions to this pattern, among which the gene pair mu*Rdr1*B and mu*Rdr1*G indicates a very recent duplication event, as the two genes are almost identical. Subclusters IIIa and IIIb harboring genes from *Malus* (IIIa) and *Prunus* (IIIb) indicate phylogenetic relationships similar to the *Rosa* and *Fragaria* subclusters in that they only include genes from one of the species, which indicates that the genes evolved after *Prunus* and *Malus* diverged. The shorter average branch lengths of these clusters compared with all other subclusters indicates that these two groups of genes are the ones that evolved most recently.

### Analysis of evolutionary rates

Enhanced rates of nucleotide diversity are a major factor in the evolutionary dynamics of NB-LRR resistance genes [46]. We therefore analyzed both TNL and non-TNL genes across rose, strawberry, apple and peach. In line with the topology of the phylogenetic tree, low values for nucleotide diversity were observed among the Rosoideae (*Rosa* and *Fragaria*) compared with the other *Rosaceae* (*Malus* and *Prunus*, Figure 4). However, if we consider the higher rates of evolution among the TNL compared with the non-TNL genes, it is somewhat surprising how closely related the *Fragaria* TNL genes are to the *Rosa* TNL genes (Figure 4). This emphasizes the very close phylogenetic relationship between roses and strawberries, which belong to the same subfamily and the same supertribe of the *Rosaceae*, which is also reflected in the high degree of macrosynteny found in their chromosome structure [42,43]. Although the *Rosaceae* TNL genes seem to be of relatively recent origin, their nucleotide diversity is more than two-fold higher when compared with the values for the cluster of flanking genes and an arbitrarily chosen gene (Figure 5). This has been observed in several NBS-LRR gene clusters and most likely reflects selective advantages due to higher rates of sequence evolution, which accelerate the evolution of new resistance specificities [46]. For many R-gene clusters, positive selection, indicated by Ka/Ks ratios of greater than 1.0, particularly in the LRR regions, has been postulated [3,23,47,48]. We also found increased Ka/Ks ratios in the LRR regions of rose *Rdr1* TNLs. However, in contrast to other studies in which a dramatic increase of Ka/Ks values has been observed in LRR regions, we found only moderate increases in the LRR region. This difference might be due to the overall lower rate of sequence evolution observed in perennial plants because of more recent duplications. Alternatively it might be caused by a lower rate of positive selection on the rose *Rdr1* gene family. There is initial evidence that the *Rdr1*-TNL cluster includes several resistance genes against black spot and that the evolution of new pathogenic races in black spot is slow due to mostly asexual reproduction and low gene flow [49].

## Conclusions

Our analyses indicate illegitimate recombination, gene conversion, unequal crossing over, indels, point mutations and transposable elements as mechanisms involved in the evolution of the *Rdr1* locus in rose. It is well documented that similar factors play a role in resistance gene diversity in several annual plant species. Therefore, the diversifying mechanisms associated with the *Rdr1* locus of the perennial, clonally propagated rose are principally comparable to those of annual plant species, although the *Rdr1* TNLs are further characterized by recent duplication.

Analyses of other TNL-type resistance genes in *Rosaceae*, for example, the recently cloned *Ma* gene from *Prunus cerasifera*[50], may provide additional information if the pattern of *Rdr1*-TNL evolution is universal for all TNL genes of perennials or specific to the rose *Rdr1* alone.

The *Rdr1* locus is highly conserved within the genus *Rosa* and somewhat less conserved between *Rosa* and *Fragaria*; nevertheless, the synteny is disturbed when compared with *Malus* and *Prunus*, possibly due to recombination and chromosome translocation aided by transposable elements.

In the Rosoideae, TNL gene diversification occurred before and after the split of *Rosa* and *Fragaria*. A similar phenomenon took place in the Spiraeoideae between *Prunus* and *Malus* TNL genes, indicating that most TNL genes in the *Rosaceae* arose relatively recently.

## Methods

DNA sequence viewing, editing and basic manipulations were performed using Bioedit [51].

### Sequencing of the *R. rugosa* BAC clones

In *R. rugosa*, sequences orthologous to the *Rdr1* region have been located in four overlapping BAC clones (31C14, 95G17, 78F5 and 35D6) [34]. Sequencing of the four overlapping *R. rugosa* BAC clones was performed following the procedures described in Terefe-Ayana et al. [35], with a few modifications. *Escherichia coli* DH10B cells carrying the BAC clones were delivered in stab agar to Cogenics (Cogenics Ltd, Morrisville, NC) for 454 FLX sequencing with 50% of a full run. The sequences were automatically clipped for adaptor and primer sequences and de novo assembled with Newbler assembler software by Cogenics (Cogenics Ltd, Morrisville, NC). Because the BAC clone sequences did not completely assemble into a single contig, subclones were generated from DNA of each BAC clone as described in Terefe-Ayana et al. [35] and Sanger sequenced using commercial sequencing services.

### Assembly and gene prediction

The sequences generated via Sanger sequencing and the first contigs generated using 454 sequencing were assembled with SeqMan (DNASTAR, Madison, WI) and ContigExpress (Invitrogen, La Jolla, CA). Gene prediction and annotation based on the completely assembled sequence was carried out using the gene prediction program FGENESH (http://www.softberry.com). To identify coding sequences in the contig and determine sequence similarity, the whole sequence was fragmented in silico into 1-kb fragments with 200-bp overlaps by the EMBOSS program Splitter (http://emboss.bioinformatics.nl/) and subjected to BLASTn and BLASTx searches [52] against the GenBank database. Domains among the putative protein coding genes were analyzed using Pfam version 23.0 (http://pfam.sanger.ac.uk/) and SMART 6 (http://smart.embl-heidelberg.de/). Sequences that were identical to a known gene in GenBank were assigned that gene name.

### Origin of strawberry, apple and peach sequences

The complete genomic sequences of apple, peach and strawberry were downloaded from the Genome Database for *Rosaceae* (http://www.rosaceae.org). Regions orthologous to the *Rdr1* locus and flanking genes were identified using local BLAST searches with the help of Bioedit [51]. Gene predictions for apple, peach and strawberry provided by the respective authors of the genome sequences [53-55] were employed directly, with some additional predictions made using FGENESH (http://www.softberry.com).

### Alignments

The complete contig sequences of *R. multiflora*[35], *R. rugosaFragaria vescaMalus x domestica* and *Prunus persica* were aligned using GATA [56] with the default parameters, then compared to determine patterns of gene clusters, the position and orientation of genes and the absence or presence of certain sequence regions.

The predicted *Rdr1* homologs (TNL homologues) and their flanking genes from each contig of *R. multiflora, R. rugosa*, strawberry, peach and apple were aligned using ClustalW with the default options [57]. For the TNL homologues, alignments were carried out for the open reading frame (ORF) and the derived amino acid sequences of the complete gene and separately for each of the TIR, NBS, LRR, exon and intron regions. Alignment of the flanking genes was performed using the complete gene sequences.

### Phylogenetic analyses

For the aligned TNL homologues from the five *Rosaceae* species, phylogenetic trees were constructed in MEGA5 using the Maximum Likelihood (ML) method based on the Kimura 2 parameter model [58,59]. Phylogenetic analysis of the amino acid sequence was performed using the Jones-Taylor-Thornton (JTT) matrix-based model in MEGA5. Initial trees for the heuristic search were obtained automatically with MEGA 5. The tree topology was tested via a bootstrap analysis with 500 replicates. The majority rule bootstrap consensus tree was taken to represent the evolutionary history of the taxa analyzed. Branches corresponding to partitions reproduced in less than 50% bootstrap replicates were collapsed.

### Analyses of Ka/Ks ratios

The ratio between the nonsynonymous nucleotide substitutions per nonsynonymous site (Ka) and synonymous nucleotide substitutions per synonymous site (Ks) was evaluated for TIR, NBS and LRR domains separately. The TIR, NBS and LRR domains were determined based on Pfam v23 (http://pfam.sanger.ac.uk/). The amino acid sequences of the different protein domains were aligned in MEGA5 using ClustalW and employed to guide the corresponding cDNA sequence alignment. The resulting cDNA alignments were used to calculate Ka and Ks with DnaSP [60] following Nei and Gojobori [36]. The selection pattern was characterized by the ratio of Ka to Ks substitution, in which Ka/Ks > 1 indicates positive selection or Darwinian adaptive evolution, Ka/Ks < 1 indicates purifying or stabilizing selection, and Ka/Ks = 1 indicates neutral evolution [61].

### Analysis of gene conversion and illegitimate recombination

The aligned TNL homologues and flanking genes from the five *Rosaceae* species were analyzed for DNA polymorphisms using π applying the Jukes and Cantor correction with DnaSP [60]. This DNA polymorphism analysis indicates the average number of nucleotide substitutions per site between two sequences, and the nucleotide diversity value is the average of all comparisons.

Recombination and sequence exchange between *Rdr1* homologues from *R. multiflora* and *R. rugosa* was determined with the programs Geneconv [39] and DnaSP [60]. The default parameters were used in both the Geneconv and DnaSP analyses. Events detected with Geneconv were examined and confirmed visually.

## Competing interests

The authors declare that they have no competing interests.

## Authors’ contributions

TD conceived and designed the experiments. DTA performed BAC subclone preparation, sequencing and assembly. DTA, HK and TD performed sequence analyses, database mining and drafted the manuscript. HK and ML contributed to and edited the manuscript. All authors have read and approved the manuscript.

## Supplementary Material

Additional file 1 ** Nucleotide sequence alignment of the *Rdr1 *region from *R. multiflora *with strawberry.** Similar sequences in the same orientation are connected by black lines. Similar sequences in reverse orientation are connected with red lines. Partial gene annotation of the *Rdr1*-TNL cluster and its flanking genes is shown for *R. multiflora*. GATAligner with default parameters was used for the alignment. GATAPlotter parameters with min: 1E^-5^ were used to plot the graph. Click here for file

Additional file 2 ** Nucleotide sequence alignment of *R. multiflora *and peach.** Similar sequences are connected by black lines. Similar sequences in reverse orientation are connected with red lines. Partial gene annotation of *Rdr1*-TNL cluster and its flanking genes is shown for *R. multiflora*. The *Rdr1*-TNL cluster and its flanking genes are split and located in two different linkage groups. GATAligner with default parameters was used for the alignment. GATAPlotter parameters with min: 1E^-5^ were used to plot the graph. Click here for file

Additional file 3 ** Nucleotide sequence alignment of *R. multiflora *and apple.** Similar sequences are connected by black lines. Similar sequences in reverse orientation are connected with red lines. Partial gene annotation of the *Rdr1*-TNL cluster and its flanking genes is shown for *R. multiflora*. The *Rdr1*-TNL cluster and its flanking genes are split and located on two different linkage groups in apple. GATAligner with default parameters was used for the alignment. GATAPlotter parameters with min: 1E^-5^ were used to plot the graph. Click here for file

Additional file 4 ** Nucleotide sequence alignment of *R. multiflora *and *M. truncatula. *** Similar sequences are connected by black lines. Similar sequences in reverse orientation are connected with red lines. Partial gene annotation of the *Rdr1*-TNL cluster and its flanking genes is shown for *R. multiflora*. In *M. truncatula* few TNL genes are shown without having the flanking genes. GATAligner with default parameters was used for the alignment. GATAPlotter parameters with min: 1E^-5^ were used to plot the graph. Click here for file

Additional file 5 ** Nucleotide sequence alignment of *R. multiflora *and *A. thaliana. *** Similar sequences are connected by black lines. Similar sequences in reverse orientation are connected with red lines. Partial gene annotation of the *Rdr1*-TNL cluster and its flanking genes is shown for *R. multiflora*. In *A. thaliana* few TNL genes are shown without having the flanking genes. GATAligner with default parameters was used for the alignment. GATAPlotter parameters with min: 1E^-5^ were used to plot the graph. Click here for file

Additional file 6 ** Nucleotide diversity (π) between TNL domains of *R. multiflora *,*R. multiflora *and *R. rugosa *, as well as *R. multiflora *,*R. rugosa *and*Fragaria. *** A significant difference in mean nucleotide diversity can be seen between TIR and NBS/LRR in *R. multiflora* and *R. rugosa* but is masked in the comparison between the three species, *R. multiflora*, *R. rugosa* and *Fragaria*. Click here for file
