# Supplementary figures and images for: Evolution of the Rdr1 TNL-cluster in roses and other Rosaceous species
Source: BMC Genomics. 2012 Aug 20;13:409. doi: 10.1186/1471-2164-13-409 (PMC3503547; doi:10.1186/1471-2164-13-409)

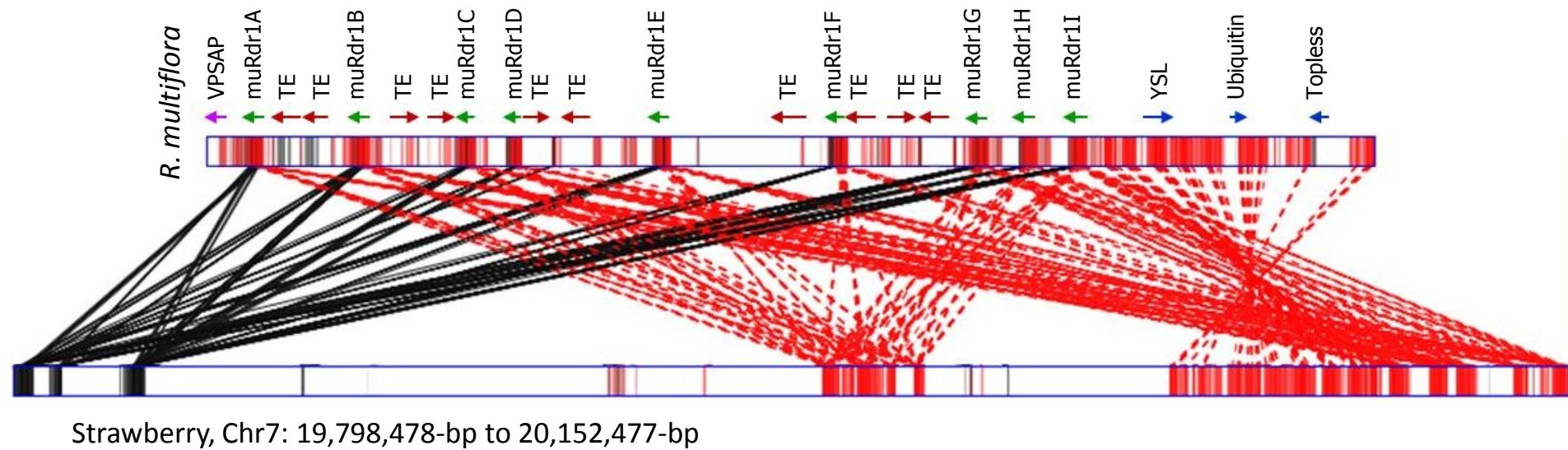

**Additional file 1**

Supplement: Additional file 1 — Nucleotide sequence alignment of the Rdr1 region from R. multiflora with strawberry. Similar sequences in the same orientation are connected by black lines. Similar sequences in reverse orientation are connected with red lines. Partial gene annotation of the Rdr1-TNL cluster and its flanking genes is shown for R. multiflora. GATAligner with default parameters was used for the alignment. GATAPlotter parameters with min: 1E-5 were used to plot the graph. [file 1471-2164-13-409-S1.pdf]

Peach, scf2: 26,050,000 bp to 26,110,000 bp

*R. multiflora*

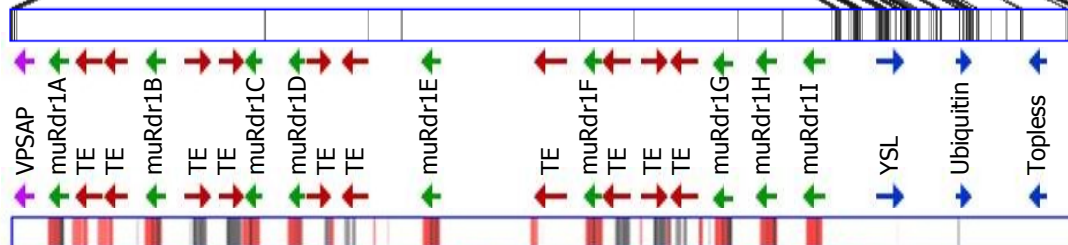

Peach, scf8: 2,050,000 bp to 2,510,000 bp

**Additional file 2**

Supplement: Additional file 2 — Nucleotide sequence alignment of R. multiflora and peach. Similar sequences are connected by black lines. Similar sequences in reverse orientation are connected with red lines. Partial gene annotation of Rdr1-TNL cluster and its flanking genes is shown for R. multiflora. The Rdr1-TNL cluster and its flanking genes are split and located in two different linkage groups. GATAligner with default parameters was used for the alignment. GATAPlotter parameters with min: 1E-5 were used to plot the graph. [file 1471-2164-13-409-S2.pdf]

Apple: chr1: 35,200,000 bp to 35,294,999 bp

*R. multiflora*

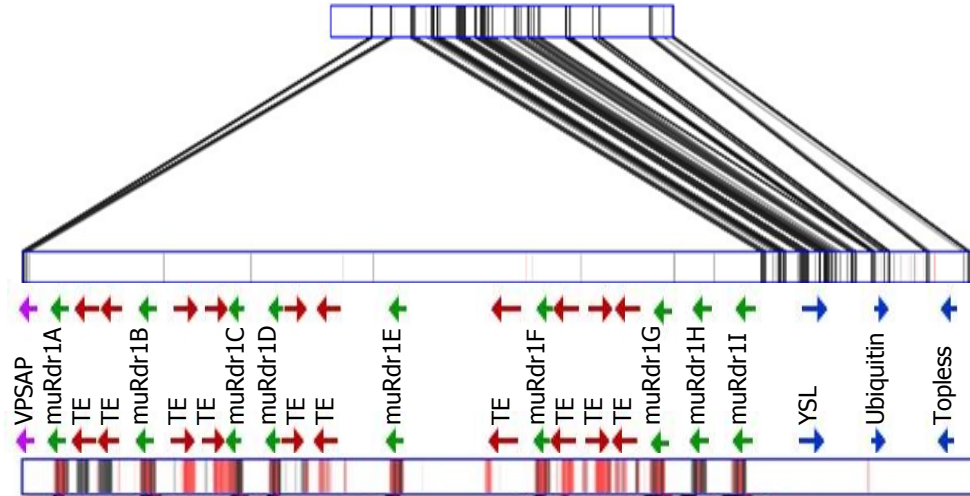

Apple: chr15: 41,166,396 bp to 41,719,891 bp

**Additional file 3**

Supplement: Additional file 3 — Nucleotide sequence alignment of R. multiflora and apple. Similar sequences are connected by black lines. Similar sequences in reverse orientation are connected with red lines. Partial gene annotation of the Rdr1-TNL cluster and its flanking genes is shown for R. multiflora. The Rdr1-TNL cluster and its flanking genes are split and located on two different linkage groups in apple. GATAligner with default parameters was used for the alignment. GATAPlotter parameters with min: 1E-5 were used to plot the graph. [file 1471-2164-13-409-S3.pdf]

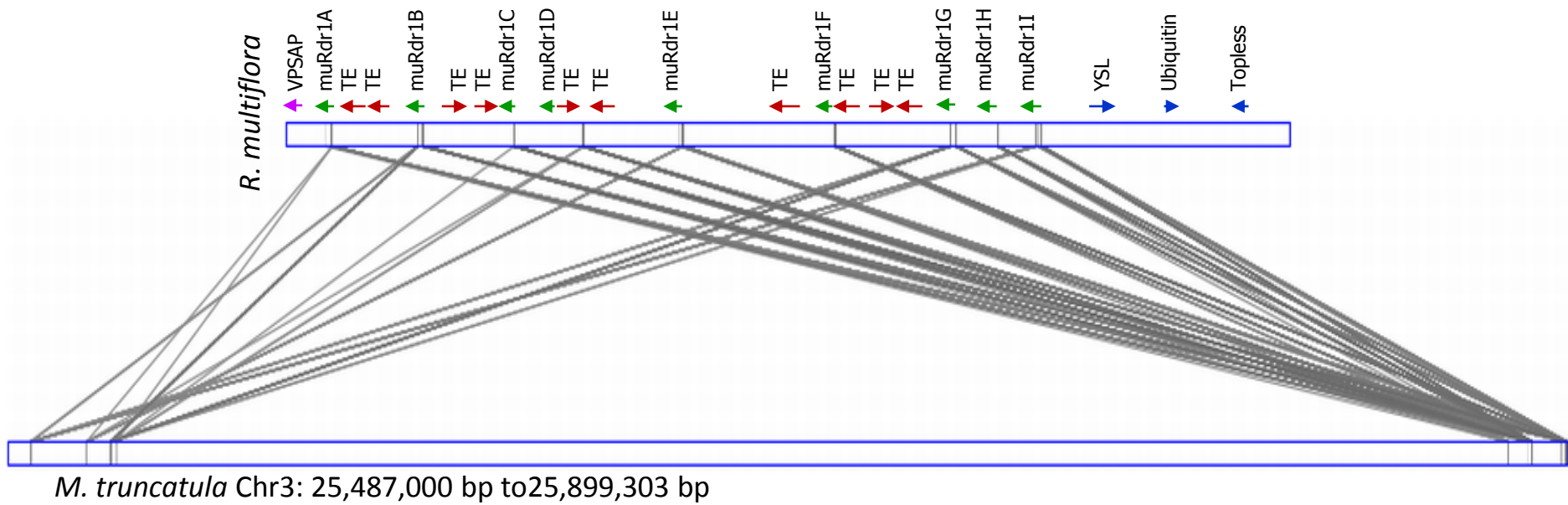

**Additional file 4**

Supplement: Additional file 4 — Nucleotide sequence alignment of R. multiflora and M. truncatula. Similar sequences are connected by black lines. Similar sequences in reverse orientation are connected with red lines. Partial gene annotation of the Rdr1-TNL cluster and its flanking genes is shown for R. multiflora. In M. truncatula few TNL genes are shown without having the flanking genes. GATAligner with default parameters was used for the alignment. GATAPlotter parameters with min: 1E-5 were used to plot the graph. [file 1471-2164-13-409-S4.pdf]

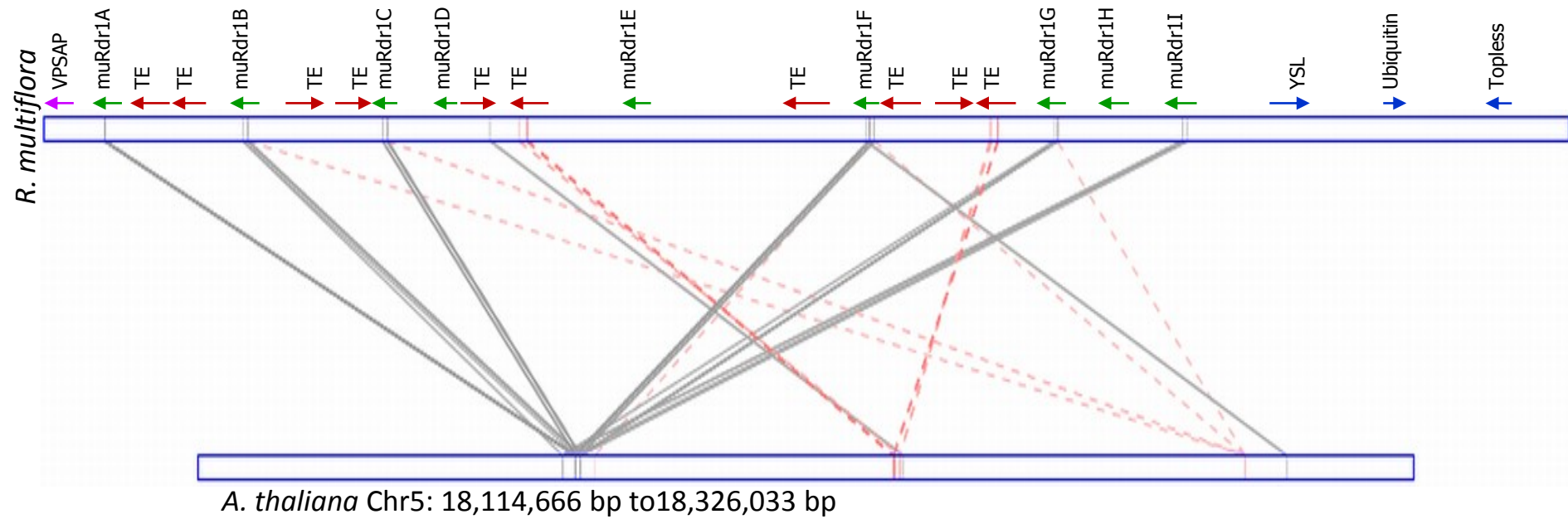

**Additional file 5**

Supplement: Additional file 5 — Nucleotide sequence alignment of R. multiflora and A. thaliana. Similar sequences are connected by black lines. Similar sequences in reverse orientation are connected with red lines. Partial gene annotation of the Rdr1-TNL cluster and its flanking genes is shown for R. multiflora. In A. thaliana few TNL genes are shown without having the flanking genes. GATAligner with default parameters was used for the alignment. GATAPlotter parameters with min: 1E-5 were used to plot the graph. [file 1471-2164-13-409-S5.pdf]

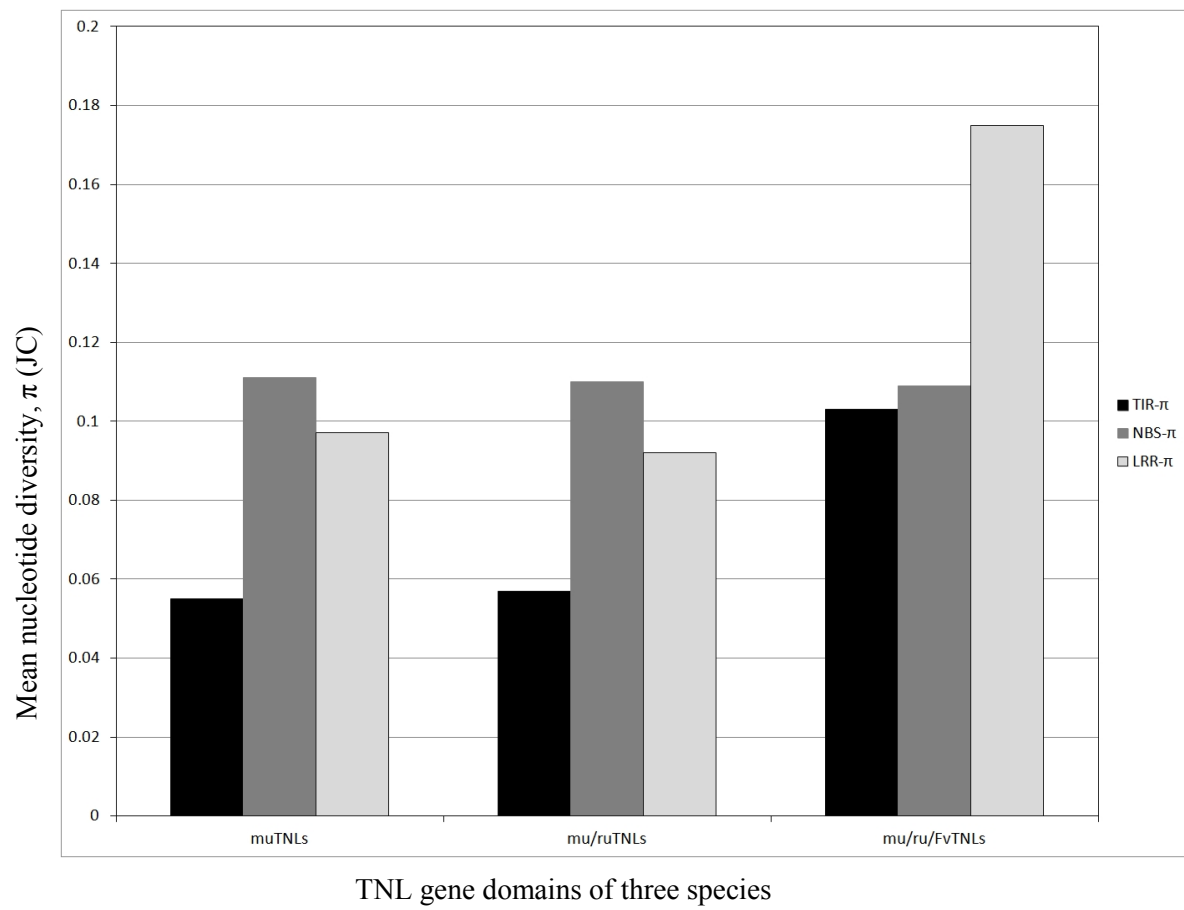

**Additional file 6**

Supplement: Additional file 6 — Nucleotide diversity (π) between TNL domains of R. multiflora ,R. multiflora and R. rugosa , as well as R. multiflora ,R. rugosa andFragaria. A significant difference in mean nucleotide diversity can be seen between TIR and NBS/LRR in R. multiflora and R. rugosa but is masked in the comparison between the three species, R. multiflora, R. rugosa and Fragaria. [file 1471-2164-13-409-S6.pdf]
